# Prevalence of irritable bowel syndrome in medical students: a systematic review and meta-analysis

**DOI:** 10.3389/fmed.2025.1714085

**Published:** 2025-12-15

**Authors:** Jhosmer Ballena-Caicedo, Fiorella E. Zuzunaga-Montoya, Lupita Ana Maria Valladolid-Sandoval, Mario J. Valladares-Garrido, Carmen Inés Gutierrez De Carrillo, Darwin A. León-Figueroa, Víctor Juan Vera-Ponce

**Affiliations:** 1 Facultad de Medicina (FAMED), Universidad Nacional Toribio Rodríguez de Mendoza de Amazonas (UNTRM), Chachapoyas, Peru; 2 Universidad Continental, Lima, Peru; 3 Escuela de Medicina Humana, Universidad Señor de Sipán, Chiclayo, Peru; 4 Facultad de Medicina Humana, Universidad de San Martín de Porres, Chiclayo, Peru; 5 EpiHealth Research Center for Epidemiology and Public Health, Lima, Peru

**Keywords:** irritable bowel syndrome, medical students, meta-analysis, prevalence, public Health

## Abstract

**Introduction:**

The high prevalence of Irritable Bowel Syndrome (IBS) in university populations, especially among medical students, raises concerns about its impact on health and academic performance.

**Objective:**

To determine the prevalence of IBS in medical students through a systematic review (SR) and meta-analysis.

**Methodology:**

An exhaustive search was conducted in PubMed, Scopus, Web of Science, and Embase. A meta-analysis was performed to combine the overall prevalence, a sensitivity analysis to evaluate the robustness of the estimates, and meta-regressions to explore the influence of variables such as publication year and Rome criteria (III and IV).

**Results:**

Forty-three studies were included: 25 studies (*n* = 13,055) used Rome III criteria and 19 studies (*n* = 6,401) used Rome IV criteria. IBS prevalence was 22.54% (95% CI: 17.51–28.01, I^2^ = 98%) with Rome III and 16.75% (95% CI: 12.49–21.49, I^2^ = 96%) with Rome IV, with substantial heterogeneity across studies. Prevalence was higher in studies using probabilistic sampling and among women. Regional analysis showed variation across WHO geographic regions (ranging from 10% to 25%), though high heterogeneity persisted within all subgroups (I^2^ > 88% in most cases). Meta-regressions showed no temporal trend.

**Conclusion:**

Irritable Bowel Syndrome is significantly prevalent among medical students, with substantial variability across studies driven by methodological factors (diagnostic criteria and sampling design) and population characteristics (sex and geographic region). High heterogeneity persisted across all subgroup analyses, indicating that local contextual factors are more influential than the broad categories examined. Probabilistic sampling protocols and standardized diagnostic criteria are recommended, as is the implementation of prevention and early management interventions in academic settings.

## 1 Introduction

Irritable Bowel Syndrome (IBS) represents one of the most frequent functional gastrointestinal disorders, characterized by chronic abdominal pain and alterations in bowel habits in the absence of detectable structural abnormalities. According to a recent meta-analysis, the global prevalence of IBS ranges between 4% and 9%, depending on the diagnostic criteria employed, constituting a significant clinical and public health challenge (1). In particular, the medical student population may present an elevated risk of IBS due to factors such as academic stress and lifestyle changes, necessitating specific evaluation (2).

Furthermore, a considerable impact has been reported at the individual level, as IBS can trigger absenteeism, decreased academic performance, and deterioration in quality of life (3). This condition entails increased demand for medical services and resource utilization in the healthcare domain. At the same time, in socioeconomic terms, it can result in indirect costs derived from reduced productivity (4). Given the complexity of these factors, the need for an updated synthesis of evidence that allows an understanding of the real magnitude of the problem in medical students becomes evident.

Despite the growing number of studies, notable knowledge gaps persist regarding the actual prevalence of IBS in medical students, partly due to the variability of diagnostic methods employed and cultural and geographical differences in case definition (5). Some investigations have reported percentages significantly higher than the global average. In contrast, others find no substantial differences compared to the general population, generating controversy about the true magnitude of this group (6). Likewise, methodological limitations have been identified, such as the use of small or non-representative samples, lack of standardization in the application of diagnostic criteria (Rome III vs. Rome IV), and heterogeneity in the evaluation of psychological and environmental factors (5).

For this reason, the main objective of the present systematic review (SR) will be to determine the prevalence of IBS in the medical student population. Achieving this objective is highly relevant from a scientific perspective, as it will clarify the epidemiology of IBS in a key group and from a clinical standpoint by guiding the implementation of preventive measures and focused interventions.

## 2 Methodology

### 2.1 Study design

An SR with meta-analysis was conducted, following the recommendations of the PRISMA (Preferred Reporting Items for Systematic Reviews and Meta-Analyses) guidelines adapted for prevalence studies (7) (Supplementary Material 1). Additionally, the MOOSE (Meta-analysis of Observational Studies in Epidemiology) guidelines were considered to strengthen the quality of the synthesis of available evidence.

### 2.2 Information search

The search was conducted in MEDLINE/PubMed, Scopus, Web of Science (which includes the SciELO catalog), and Embase, following the methodological recommendations of the Cochrane guidelines for systematic reviews. The selection of these databases was justified by their broad thematic coverage, international scope, and relevance to the field of health sciences. For the development of the search strategy, MeSH (Medical Subject Headings) terms and free keywords were employed and selected based on the condition of interest, target population, and type of study. These terms were grouped and adjusted according to the syntax of each database to cover all possible variants of the condition (e.g., “Irritable Bowel Syndrome”), the population (e.g., “medical students”), and research designs focused on prevalence.

Combining terms was performed through Boolean operators (AND, OR, NOT), incorporating synonyms and related terms to maximize search sensitivity and reduce the risk of omitting relevant studies. The search covered up to March 6, 2025. A basic strategy was created in PubMed, which was subsequently adapted to other databases to maintain consistency. The complete strategy, with specific details of fields and filters applied in each repository, is available in Supplementary Material 2.

### 2.3 Selection criteria

Observational studies, preferably cross-sectional that reported data on the prevalence of IBS in the population above, were included, applying validated diagnostic criteria, specifically only ROME III and IV. Studies were required to present prevalence results clearly or with the possibility of data extraction in English or Spanish. Likewise, investigations from different geographic regions were considered, provided they met quality criteria and methodological relevance and offered consistent definitions of the studied condition.

All studies that did not provide prevalence data were excluded, as well as letters to the editor, case reports, narrative or bibliographic reviews, and studies that did not explicitly describe the diagnostic methods employed. Investigations that included populations with specific characteristics or different from the main population of interest were also not accepted, nor were studies in which the condition was defined differently from that used in the accepted criteria. In case of detecting duplicates or multiple reports of the same study, the one offering more complete or updated data was considered.

### 2.4 Study selection process

The search results were imported into Rayyan software, where duplicate references were eliminated automatically and manually. Subsequently, two independent reviewers blindly examined the titles and abstracts to identify studies that might meet the inclusion criteria. Once this first screening phase was completed, the full texts of potentially eligible articles were reviewed to confirm their definitive inclusion in the review.

In cases where discrepancies arose between reviewers, a discussion and consensus mechanism was applied, and if the discrepancy persisted, the opinion of a third reviewer was requested. This process ensured impartiality and coherence in evaluating the relevance of each study. The number of discarded references and reasons for exclusion were systematically recorded, and all steps followed for the final selection of studies were subsequently reflected in the corresponding PRISMA flow diagram.

### 2.5 Data extraction

Two researchers independently extracted data from the finally selected studies using a standardized form in Excel 2023. This form included bibliographic information [author(s) and year of publication], study characteristics (design, country or region, and data collection period), and details of the evaluated population (sample size, sex, and age distribution). Sampling, recruitment methods, and diagnostic criteria or definitions used to determine the condition of interest were also recorded.

Once extraction was completed, each reviewer verified the data with the original documentary base to ensure information fidelity. If discrepancies between reviewers were detected, differences were discussed, and, if necessary, a third researcher intervened to reach a consensus. Data on the main outcome measures, in this case, the prevalence of the condition, and other relevant secondary results, such as the presence of associated factors or subgroups of interest, were also extracted, provided they were clearly reported in the original publication.

### 2.6 Risk of bias assessment

The risk of bias assessment was independently carried out by two researchers, using the Munn et al. tool (8) specifically designed for prevalence studies. This tool considers several domains, including sample representativeness and selection, quality of measurement of the variable of interest, transparency in the definition of the target population, and coherence in the presentation of results. For each of these domains, a classification ranging from “low risk,” “uncertain risk,” or “high risk” of bias is assigned, which contributes to an overall score that synthesizes the level of methodological quality of each study.

Any discrepancy in the reviewers’ rating was resolved through discussion, and if discord persisted, a third researcher was consulted to reach a consensus. To integrate these assessments into subsequent analysis, the overall risk of bias score was taken into account when interpreting findings and their consistency, as well as in possible sensitivity analyses in case of performing a meta-analysis. In this way, a rigorous and systematic approach was guaranteed that allowed for assessing the solidity and reliability of the available evidence.

### 2.7 Statistical analysis

Quantitative analyses were performed using R statistical software version 4.2.2, considering only those studies that reported sufficient data on IBS prevalence, including the total number of participants (*n*) and the number of cases (r). This information was essential to calculate prevalence estimates reliably, integrating data in a standardized manner. No minimum number of studies was pre-specified as a threshold for conducting meta-analysis; this decision was guided by the volume of available evidence identified through systematic search. In addition, specific packages such as meta and metafor were used, which offer specialized functions for handling proportions and performing random effects models.

For the execution of the meta-analysis, the metaprop function of the meta package was employed, configuring the transformation of proportions using the Freeman-Tukey method (sm = “PFT”). This transformation stabilizes the variance associated with proportions, improving the precision of estimates in prevalence studies. Confidence intervals (CI) were calculated using the Clopper-Pearson method (method.ci = “CP”), which provides exact binomial confidence intervals without relying on normal approximation assumptions. This method is particularly appropriate for prevalence meta-analysis as it maintains correct coverage probability even with extreme proportions (near 0% or 100%) or small sample sizes, unlike methods based on normal approximation (e.g., Wald intervals) that can produce intervals outside the [0,1] range or underestimate uncertainty. Given the expected heterogeneity in populations and methods of each study, a random effects model was chosen using the DerSimonian and Laird method (method.tau = “DL”), also correcting standard errors with the Hartung-Knapp approximation (hakn = TRUE) to reinforce the robustness of estimates.

Heterogeneity was routinely evaluated using the I^2^ statistic and Cochran’s Q test, the results of which were automatically generated through the metaprop function. To visualize the results, forest plots were created for each synthesized estimate, showing the individual prevalence of each study along with the overall prevalence, one according to Rome III and another according to Rome IV. Subgroup analyses were conducted to explore potential sources of heterogeneity in IBS prevalence. For both Rome III and Rome IV criteria separately, studies were stratified by sex (women vs. men), sampling design (probabilistic vs. non-probabilistic), country, and WHO geographic region (African Region, Region of the Americas, Eastern Mediterranean Region, European Region, South-East Asia Region, and Western Pacific Region). Pooled prevalence estimates were calculated within each subgroup using random effects models.

Publication bias was assessed visually using funnel plots and statistically through Egger’s regression test for small-study effects, implemented using the metabias function from the meta package. The test examines whether smaller studies (with larger standard errors) show systematically different effect estimates than larger studies, which may indicate publication bias or other sources of small-study effects.

Additionally, meta-regressions were carried out to investigate the influence of continuous variables, such as the year of publication and the diagnostic criteria used. These models were executed using the rma function of the metafor package, which allows adjusting mixed effects models by assigning weights inversely proportional to the variance of each study. Meta-regression results, including regression coefficients (β), standard errors, *p*-values, and R^2^ values (proportion of between-study variance explained by the predictor), were calculated to quantify temporal trends in IBS prevalence. To illustrate these analyses, bubble plots were used, where the size of each bubble represented the statistical weight of the study.

Furthermore, to geospatially represent the distribution of prevalence, a world map stratified by country and sex was developed, offering a global perspective on the variability of IBS in different contexts.

## 3 Results

### 3.1 Article selection

**FIGURE 1:**
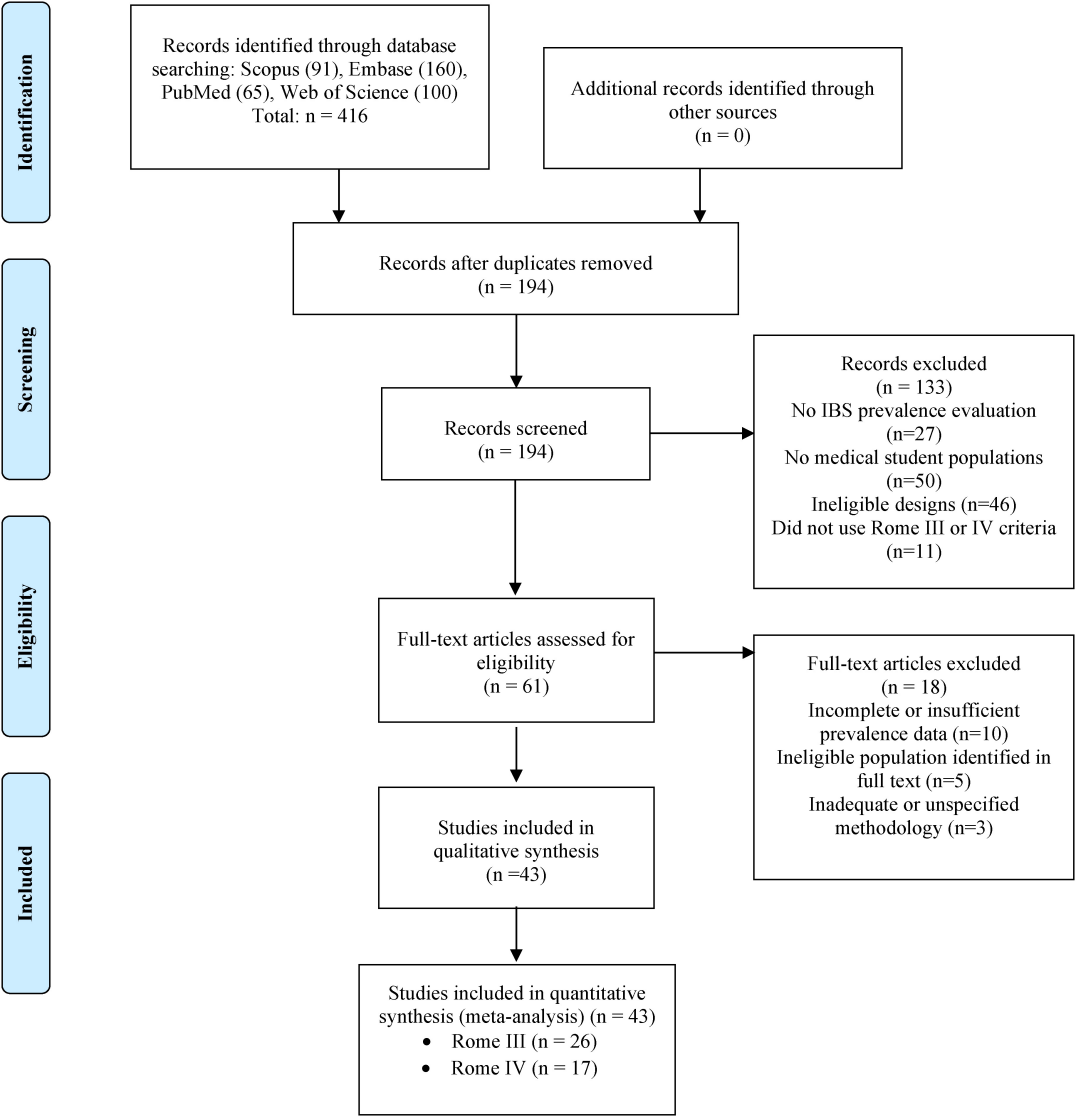
Flowchart of study selection.

The PRISMA flow diagram illustrates the systematic process of study selection. Initially, 416 records were identified through the four aforementioned databases. After removing duplicates, 194 unique records were obtained for review. Of these, 133 were excluded for various reasons: not evaluating IBS prevalence (*n* = 27), not including medical students (*n* = 50), ineligible designs (*n* = 46), or not using Rome III/IV criteria (*n* = 11). Of the 61 articles evaluated in full text, 18 more were excluded due to insufficient data (*n* = 10), the ineligible population identified in full text (*n* = 5), or inadequate methodology (*n* = 3). Finally, 43 studies were included in the qualitative synthesis and the meta-analysis (9–51) (Figure 1).

### 3.2 Main characteristics

The 43 included studies span a publication period between 2011 and 2024, with a notable increase in research from 2020 (almost two-thirds of the total) (Supplementary Material 3). These works come from multiple WHO regions and geographic areas: Eastern Mediterranean (Saudi Arabia, Egypt, Jordan, United Arab Emirates, Oman, Iran, Yemen, Pakistan, Tunisia, Sudan) (9, 17, 19, 20, 22, 28, 30–33, 35, 37, 41, 49, 51), Western Pacific and South-East Asia (Korea, China, Malaysia, India, Bangladesh) (9, 11–13, 16, 25, 36, 40, 45, 47), Americas (Peru, Colombia, Mexico, Canada, Puerto Rico) (10, 14, 18, 21, 27, 39, 42, 48, 50), Europe (Slovakia, Malta) (18, 43), and Africa (Benin) (22) Saudi Arabia had the highest number of studies (*n* = 8) (17, 28, 30–33, 41, 46), followed by Peru (*n* = 5) (14, 21, 42, 48, 50) and China (*n* = 3) (13, 16, 36), demonstrating broad geographic representation. Most publications were in English, with a smaller percentage in Spanish from studies conducted in Spanish-speaking countries.

All studies opted for a cross-sectional design. Sampling methods varied between probabilistic and non-probabilistic; some studies implemented simple or stratified sampling strategies, while others resorted to convenience selection. Regarding sample size, great heterogeneity was evidenced, with figures ranging between 100 and 2,739 participants, with most studies having samples of between 300 and 600 students.

The target population included medical students from different universities and faculties, generally covering ages between 18 and 25 years. Most studies included both males and females, with a female proportion that, on average, was between 40% and 70%, although some works presented predominantly female samples or did not report the breakdown by sex in detail. While most focused on urban settings or universities located in large cities, some studies contemplated broader recruitments that combined students from urban and rural areas. In general terms, participants shared a similar academic profile, ranging from the first to the last year of medical school, with exclusion criteria aimed at ruling out organic gastrointestinal pathologies or alarm signs.

Regarding the risk of bias analysis, all included studies were evaluated as “low risk,” with scores ranging between 7 and 8 points on the evaluation scale. It is observed that studies that employed probabilistic sampling (13, 18, 20, 22, 29, 36, 41, 46, 49–51) systematically received a higher score (8 points), compared to those that used non-probabilistic sampling methods, which obtained 7 points. All included studies correctly applied the Rome III or IV criteria for IBS diagnosis and specifically focused on medical student populations, thus ensuring homogeneity in diagnostic criteria and target population, reinforcing the findings’ validity for this specific population (see Supplementary Material 4).

### 3.3 Meta-analysis and meta-regression of IBS prevalence–Rome III and IV

Based on the diagnostic criteria employed for the definition of IBS, a higher global prevalence was observed in studies that applied Rome III criteria (25 studies, *n* = 13,055) (9–18, 20–22, 25, 27, 31–36, 38, 39, 44, 45), with a point estimate of 22.54% (95% CI: 17.51–28.01) and a very high heterogeneity index (I^2^ = 98%). In contrast, studies based on Rome IV (19 studies, *n* = 6,401) (19, 23, 24, 26, 28–30, 40–43, 47–51) reported a prevalence of 16.75% (95% CI: 12.49–21.49), with I^2^ = 96% (Table 1).

**TABLE 1:** Results of the meta-analysis of IBS prevalence according to Rome III and IV criteria, sampling design, and sex of participants.

Criteria/Category	Number of studies	Number of participants	95% CI	I^2^
**Criteria used to define IBS**				
Rome III	25	13055	22.54 (17.51 – 28.01)	98%
Rome IV	19	6401	16.75 (12.49 – 21.49)	96%
**Sampling design**				
Rome III: probabilistic	5	4570	29.54 (12.97 – 49.51)	99%
Rome III: non-probabilistic	20	8485	20.90 (16.12 – 26.12)	97%
Rome IV: probabilistic	6	1743	20.51 (14.02 – 27.85)	96%
Rome IV: non-probabilistic	13	4658	15.11 (9.98 – 21.06)	96%
**Sex of participants**				
Rome III: women	16	5249	26.90 (20.65 – 33.63)	96%
Rome III: men	16	4775	19.74 (15.42 – 24.45)	92%
Rome IV: women	12	2116	21.61 (14.93 – 29.13)	94%
Rome IV: men	12	2088	16.18 (12.21 – 20.58)	84%
**WHO geographic region (Rome III)**				
Eastern Mediterranean Region	11	4121	24.23 (17.08 – 32.18)	97%
Region of the Americas	6	2002	24.92 (9.58 – 44.48)	99%
South-East Asia Region	3	895	13.73 (4.20 – 27.47)	96%
Western Pacific Region	5	6037	21.77 (13.08 – 31.94)	99%
**WHO geographic region (Rome IV)**				
Eastern Mediterranean Region	9	3285	21.04 (14.11 – 28.92)	96%
Region of the Americas	3	677	15.03 (11.01 – 19.55)	52%
African Region	1	315	13.97 (10.57 – 18.23)	–
European Region	2	1196	11.67 (3.37 – 23.92)	92%
Western Pacific Region	3	928	10.19 (5.20 – 16.58)	88%

**FIGURE 2:**
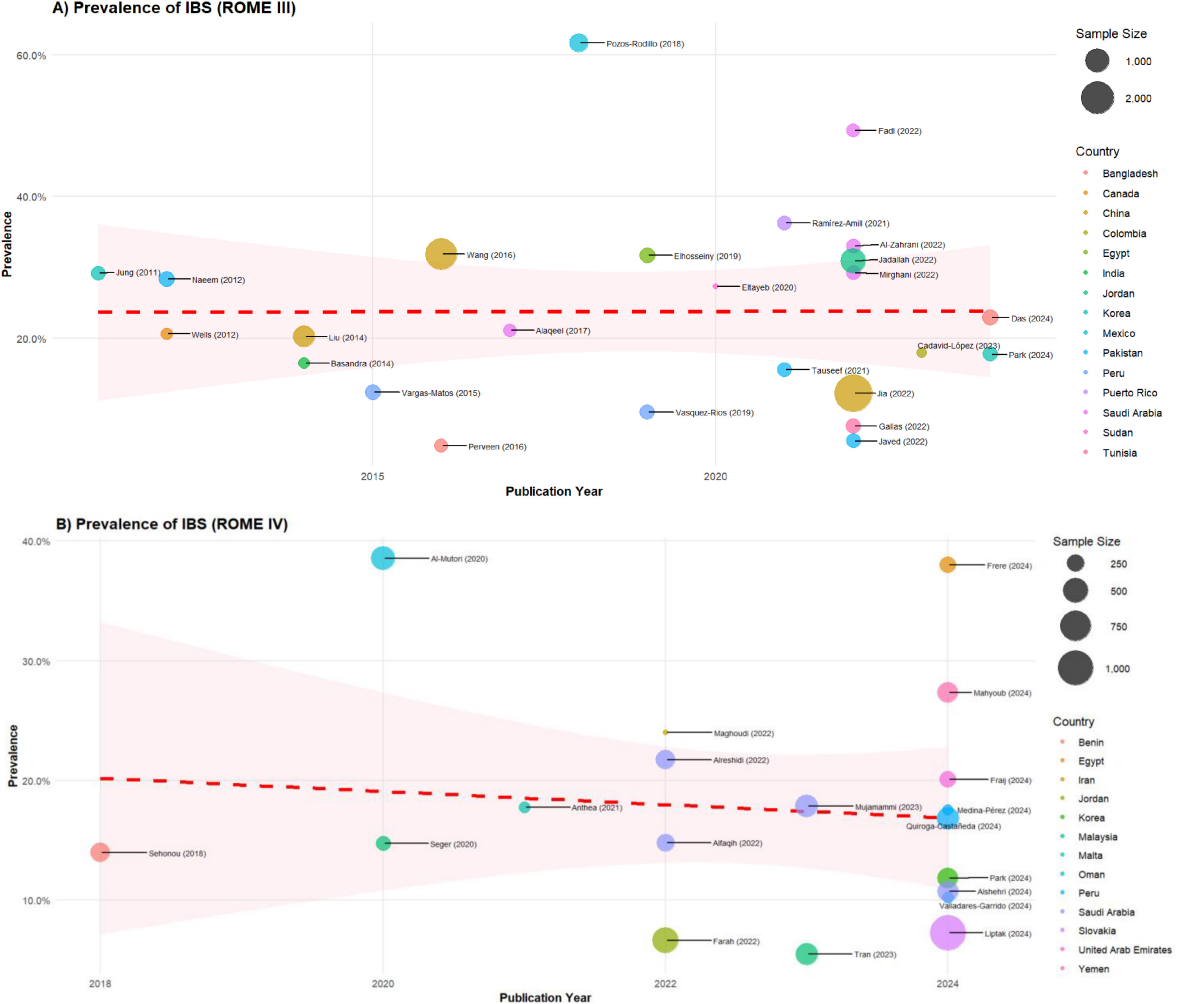
Meta-regression of IBS prevalence by year and country according to ROME III and IV. **(A)** Studies based on the Rome III criteria, **(B)** studies using the Rome IV criteria. It has been organized to facilitate a detailed analysis of each criterion, enabling a clearer understanding of the differences and trends across studies that utilized these distinct criteria.

Figure 2 presents the meta-regression analysis exploring the association between publication year and IBS prevalence for studies that used Rome III criteria (panel A) and Rome IV (panel B). For Rome III criteria (*k* = 25 studies), the regression coefficient was β = −0.0004 (SE = 0.0083, 95% CI: −0.017 to 0.016, *p* = 0.957), indicating no significant temporal trend. Similarly, for Rome IV criteria (*k* = 19 studies), the coefficient was β = −0.0080 (SE = 0.0164, 95% CI: −0.040 to 0.024, *p* = 0.627). In both models, publication year explained essentially none of the between-study variance (R^2^ = 0.0% for both criteria), and residual heterogeneity remained very high after accounting for temporal trends (I^2^res = 98.0% for Rome III; I^2^res = 94.9% for Rome IV).

### 3.4 Subgroup analysis: IBS prevalence in studies with probabilistic sampling, sex and WHO geographic region

When breaking down the results according to sampling design, studies that applied Rome III and employed probabilistic sampling (5 studies, *n* = 4,570) (13, 18, 20, 22, 36) reported the highest prevalence, 29.54% (95% CI: 12.97–49.51, I^2^ = 99%), compared to those that used non-probabilistic sampling (20 studies, *n* = 8,485), with 20.90% (95% CI: 16.12–26.12, I^2^ = 97%) (9–12, 14–17, 21, 25, 27, 31–35, 38, 39, 44, 45). A similar trend was observed in studies with Rome IV, where probabilistic sampling (6 studies, *n* = 1,743) (29, 41, 46, 49–51) showed a prevalence of 20.51% (95% CI: 14.02–27.85, I^2^ = 96%), while in non-probabilistic sampling (13 studies, *n* = 4,658) (19, 23, 24, 26, 28, 30, 37, 40, 42, 43, 45, 47, 48) the figure was 15.11% (95% CI: 9.98–21.06, I^2^ = 96%) (Table 1).

In the analysis by sex, women evaluated with Rome III criteria (16 studies, *n* = 5,249) (9, 11–13, 15–17, 20–22, 25, 27, 31, 33, 35, 36) showed a prevalence of 26.90% (95% CI: 20.65–33.63, I^2^ = 96%), compared to 19.74% (95% CI: 15.42–24.45, I^2^ = 92%) observed in men (*n* = 4,775) (9, 11–13, 15–17, 20–22, 25, 27, 31, 33, 35, 36). Under Rome IV, a similar pattern was maintained with rates of 21.61% (95% CI: 14.93–29.13, I^2^ = 94%) for women (12 studies, *n* = 2,116) (19, 23, 24, 28–30, 37, 41, 46–50) and 16.18% (95% CI: 12.21–20.58, I^2^ = 84%) in men (*n* = 2,088) (19, 23, 24, 28–30, 37, 41, 42, 46–50) (Table 1).

Regional subgroup analysis revealed variation in IBS prevalence across WHO geographic regions. For Rome III criteria, the Region of the Americas showed the highest prevalence at 24.92% (95% CI: 9.58–44.48, I^2^ = 99%), followed by the Eastern Mediterranean Region at 24.23% (95% CI: 17.08–32.18, I^2^ = 97%), the Western Pacific Region at 21.77% (95% CI: 13.08–31.94, I^2^ = 99%), and the South-East Asia Region at 13.73% (95% CI: 4.20–27.47, I^2^ = 96%). For Rome IV criteria, the Eastern Mediterranean Region had the highest pooled prevalence at 21.04% (95% CI: 14.11–28.92, I^2^ = 96%), followed by the Region of the Americas at 15.03% (95% CI: 11.01–19.55, I^2^ = 52%), the African Region at 13.97% (95% CI: 10.57–18.23), the European Region at 11.67% (95% CI: 3.37–23.92, I^2^ = 92%), and the Western Pacific Region at 10.19% (95% CI: 5.20–16.58, I^2^ = 88%). Despite this regional variation, high heterogeneity persisted within nearly all geographic subgroups (I^2^ > 88% in most regions, with the exception of the Americas under Rome IV showing I^2^ = 52%) (Table 1).

### 3.5 Meta-analysis of IBS prevalence by country according to Rome III and IV criteria

**FIGURE 3:**
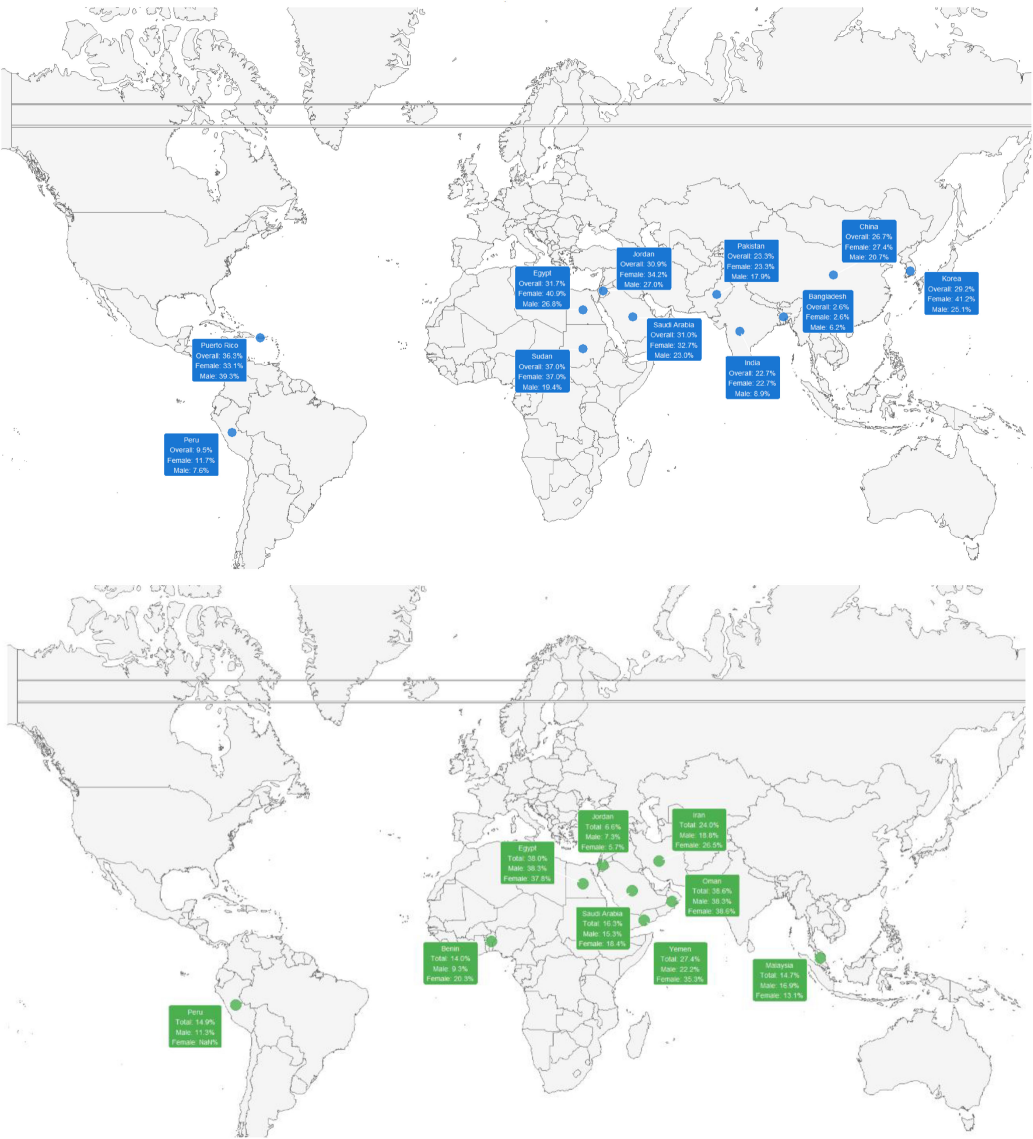
Global distribution of IBS prevalence according to Rome III (upper) and IV criteria, stratified by sex.

The upper part of Figure 3 shows the global distribution of IBS prevalence according to Rome III criteria, differentiating each country through blue boxes that include the overall prevalence, as well as the breakdown by sex. A wide variability in the reported percentages is observed, even within the same geographic region, suggesting heterogeneity in the studied population and evaluation methods. In general, certain countries in Asia and the Middle East present higher estimates than other regions, while in Latin America, prevalences tend to be located in intermediate ranges, with slight differences between men and women.

The lower part of the figure illustrates the prevalence of IBS based on Rome IV criteria, indicated with green boxes. While the geographic coverage is similar to that of studies with Rome III, in this grouping the prevalence estimates tend to be globally lower, according to the findings in the meta-analysis. Differences in the comparison by sex persist, although the values show a slightly reduced dispersion range compared to data based on Rome III. This comparison suggests that the choice of diagnostic criteria may influence the percentages observed in different regions.

#### 3.5.1 Publication bias assessment

Funnel plots were constructed to visually assess publication bias (Supplementary Material 5). Both Rome III (panel A) and Rome IV (panel B) funnel plots showed asymmetric distribution of studies, with points scattered on both sides of the central axis but not completely symmetrical. The Freeman-Tukey double arcsine transformation (*X*-axis) revealed that studies with Rome III presented greater dispersion (0.2–0.9) compared to those with Rome IV (0.3–0.7), while standard error (*Y*-axis) was similar in both sets. This pattern suggested potential heterogeneity in study characteristics rather than systematic publication bias.

To formally test for publication bias, Egger’s regression test for small-study effects was conducted. For Rome III studies, Egger’s test showed *t* = 0.27 (df = 23, *p* = 0.791), and for Rome IV studies, the test yielded *t* = 1.71 (df = 17, *p* = 0.106). Neither test reached statistical significance at the conventional α = 0.05 level, indicating no strong statistical evidence of funnel plot asymmetry attributable to publication bias. These results should be interpreted cautiously in prevalence meta-analyses, as funnel plot asymmetry may reflect genuine heterogeneity in study populations, sampling methods, and geographic settings rather than selective publication alone. The observed asymmetry is more likely attributable to methodological diversity–particularly the distinction between probabilistic and non-probabilistic sampling–and substantial regional variation in IBS prevalence rather than systematic suppression of studies with lower prevalence estimates.

## 4 Discussion

### 4.1 Main findings

The main findings of this systematic review and meta-analysis point to a higher global prevalence of IBS with Rome III criteria (22.54%) than with Rome IV (16.75%), which was maintained regardless of the sampling method. In both cases, studies that employed probabilistic designs reported higher figures (Rome III: 29.54%; Rome IV: 20.51%) than those with non-probabilistic sampling (Rome III: 20.90%; Rome IV: 15.11%). In addition, the analysis by sex revealed a higher proportion of IBS in women than in men, for both Rome III and Rome IV, with prevalences of 26.90% vs. 19.74% and 21.61% vs. 16.18%, respectively. Regional subgroup analysis showed variation across WHO geographic regions. For Rome III, prevalence ranged from 13.73% in South-East Asia to 24.92% in the Americas. For Rome IV, estimates ranged from 10.19% in the Western Pacific Region to 21.04% in the Eastern Mediterranean Region, with Africa (13.97%) and Europe (11.67%) also represented.

These estimates were characterized by notable heterogeneity (I^2^ > 90% in practically all subgroups), suggesting substantial variations in the studied populations, recruitment settings, and evaluation methods. High heterogeneity persisted within all regional subgroups (I^2^ > 88% in most cases, with the exception of the Americas under Rome IV showing I^2^ = 52%), indicating that broad geographic classification alone does not account for variability. Nevertheless, the differences observed between diagnostic criteria (Rome III vs. Rome IV), sampling schemes, distribution by sex, and regional variation constitute key points for understanding the real magnitude of the condition in medical students and guiding future research.

### 4.2 Interpretation of results

The findings of this systematic review and meta-analysis indicate an IBS prevalence ranging between 16.75% and 22.54%, depending on the diagnostic criteria applied (Rome IV or Rome III, respectively). This partially coincides with previous reviews focused on university populations or medical students. For example, Ibrahim et al. (2) indicated an even wider range of prevalence (9.3%–35.5%) in their 2016 review, although their estimation did not contemplate such an exhaustive separation by different diagnostic criteria. Similarly, Yang et al. (5), in a meta-analysis of Chinese studies, found considerable IBS figures in the university population in general, although medical schools were not differentiated from other study programs. Such variations highlight the diversity of methodologies and definitions employed, which makes direct comparison of estimates difficult, but confirms that IBS in the university setting–and particularly among medical students–constitutes a frequent problem.

When comparing the results with data on IBS prevalence in the general population, notable differences are observed. On one hand, Oka et al. (1) reported in their global review prevalences ranging between 4% and 9%, although their analysis included populations of multiple ages and cultural contexts without the specific stress that medical training entails. In contrast, the higher percentage of IBS in medical students identified in the present study may be due to factors such as academic overload, the demands of the medical curriculum, and the predisposition of these students to perceive and record functional symptoms. Likewise, the high heterogeneity (I^2^ > 90% in many analyses) emphasizes the influence of variables such as the geographical origin of students, recruitment strategies, and the adopted diagnostic criteria.

The high heterogeneity (I^2^ > 90% in many analyses) observed in this meta-analysis is expected and reflects genuine diversity across studies rather than methodological limitations. All 43 included studies were rated as having a low risk of bias (7–8 points on the Munn scale), confirming their methodological quality. We conducted a comprehensive exploration of heterogeneity sources through multiple approaches: subgroup analyses by diagnostic criteria (Rome III vs. IV), sampling methodology (probabilistic vs. non-probabilistic), sex, and WHO geographic region; geographic stratification by country; and meta-regression by publication year. These analyses revealed clinically meaningful patterns, including higher prevalence with Rome III criteria compared to Rome IV, in studies using probabilistic sampling compared to non-probabilistic designs, and in women compared to men. Meta-regression showed no temporal trend in IBS prevalence over time. However, high heterogeneity persisted across all subgroup analyses (I^2^ > 88% in most cases), reflecting inherent differences in population characteristics (age distribution, academic year, stress exposure), cultural factors (dietary patterns, healthcare-seeking behavior, stigma around gastrointestinal symptoms), and environmental contexts (urban vs. rural settings, healthcare systems, academic demands) across the 20+ countries represented. This variability should be interpreted as evidence diversity rather than analytical weakness, appropriately managed through random-effects models that account for between-study variance.

Regarding geographic distribution, higher estimates were found in some nations in Asia and the Middle East, in accordance with what was reported by Almansour (52) and Makkawy et al. (53), who found significant IBS figures in the Arab population. Regional subgroup analysis confirmed this pattern, with the Eastern Mediterranean Region showing the highest pooled prevalence for both Rome III (24.23%) and Rome IV (21.04%) criteria. For Rome III, prevalence ranged from 13.73% in South-East Asia to 24.92% in the Americas, while Rome IV estimates ranged from 10.19% in the Western Pacific to 21.04% in the Eastern Mediterranean, with additional data from Africa (13.97%) and Europe (11.67%). However, even when studies were grouped by WHO region, heterogeneity remained extremely high (I^2^ > 88% in most regions, with the exception of the Americas under Rome IV showing I^2^ = 52%), indicating that broad geographic categories do not capture the full complexity of factors driving prevalence variation. Even within the same country, prevalence may vary depending on the institution or sampling design, which explains why reviews specifically focused on Saudi Arabia, such as that of Almasary et al. (54), show a wide range of results. Cultural, dietary, and social factors could underlie these discrepancies, as well as the consideration of different subgroups of the academic community and the application of divergent diagnostic tools.

The comparison by sex in our study suggests a higher proportion of IBS in women than in men, with prevalences between 5 and 7 percentage points higher according to the Rome criteria employed. This pattern is consistent with the trend identified in previous meta-analyses that analyze the distribution of IBS according to gender (55). It is hypothesized that hormonal and psychosocial factors, added to a greater predisposition of women to seek medical attention or report their symptoms, could influence this result. However, it should be noted that in some countries the sample predominantly included women or, in others, the exact proportion of males was not specified, which could generate biases in the calculation of stratified prevalences.

Finally, the contrast between probabilistic and non-probabilistic sampling evidenced substantial differences in prevalence, being significantly higher in the former. This finding aligns with what is described in reviews that highlight the importance of representative sampling when investigating the epidemiology of functional diseases (2). Studies with probabilistic sampling tend to capture a wider range of students, avoiding self-selection and other associated biases, which could explain the higher rate of IBS observed. These results suggest the convenience of standardizing recruitment and diagnostic procedures as much as possible, promoting greater methodological rigor that allows for more accurate comparison of findings between countries and over time.

### 4.3 Implications for public health and medical education

First, the results of this systematic review indicate the importance of recognizing that IBS not only significantly affects the general population, but also presents a considerable prevalence among medical students. This implies the need to incorporate early detection and intervention programs in higher education institutions to reduce academic stress and promote healthy lifestyle habits. Such programs could include nutritional counseling, psychological support, and prevention activities promoting a comprehensive health care vision.

Second, these implications extend to curriculum design in medical schools. Given that those who suffer from IBS may present disruptive symptoms such as abdominal pain and alterations in bowel habits, the training curriculum must include subjects or modules aimed at understanding functional gastrointestinal disorders. This approach would allow future doctors to develop specific competencies to diagnose and manage IBS in their patients, favoring a holistic approach that considers psychosocial and behavioral factors.

A third aspect is the need to promote epidemiological and clinical research on IBS and other functional disorders in the university environment. Strengthening surveillance and follow-up of these symptoms among medical students through longitudinal studies and collaborative projects would provide additional evidence on risk factors, symptom patterns, and the effectiveness of different preventive and therapeutic interventions. Having more precise and contextualized data in different regions will, in turn, allow for establishing comparisons and designing more adequate public health strategies.

Finally, from a health policy perspective, the findings highlight the need to incentivize the formation of multidisciplinary teams that integrate nutritionists, gastroenterologists, psychologists, and health educators, with a view to addressing IBS preventively in universities. In this way, it would decrease the associated morbidity and the eventual expenditure on health services. In addition, raising awareness among academic authorities and local governments about the magnitude of the problem is crucial to guarantee financial, logistical, and regulatory support for this type of initiative.

### 4.4 Consequences of inaction

First, not addressing IBS in a timely manner in medical students can lead to prolonged underdiagnosis and the development of physical and mental comorbidities. Some reviews have demonstrated a relationship between functional gastrointestinal disorders and the appearance of depressive or anxiety symptoms, attributable in part to the chronicity of pain and diagnostic uncertainty (2, 4). This situation could foster unfavorable coping behaviors, such as excessive use of analgesics, self-medication, or avoidance of social and academic situations, which in turn perpetuate the deterioration of quality of life and psychological state (5, 52).

Second, inaction is associated with a potentially negative impact on future health professionals’ academic training and clinical performance. Previous studies have described how IBS can interfere with daily activities and school performance, contributing to absenteeism and lower concentration during classes and clinical rotations (2). This poses a risk to the quality of medical education, as the preparation of students–especially in advanced courses with greater clinical demands–may be affected by the constant distraction of symptoms and associated distress (1, 56). In the long term, the repercussions on the training of doctors could translate into limitations to adequately face healthcare challenges, impacting patient care.

Third, the absence of interventions aimed at preventing and treating IBS in the university environment can have repercussions on the health system, both at an economic and organizational level. According to various reviews, functional gastrointestinal disorders are related to greater use of medical resources, diagnostic tests, and specialist referrals (1, 55, 57). As these students graduate and enter professional practice, uncontrolled stress, persistent symptoms, and possible chronification of the condition could increase the risk of complications and gastrointestinal comorbidities, raising the total costs of care (52, 54). Thus, the implementation of screening programs and early management of IBS would not only benefit the comprehensive training of future doctors but could also alleviate the burden on health services in the long term.

### 4.5 Strengths and limitations

One of this systematic review’s main strengths is its bibliographic search’s breadth, which covered multiple extensive databases and a prolonged publication period. In addition, only studies with Rome III and IV were selected. Finally, meta-analyses and subgroup analyses (diagnostic criteria, sampling, sex) provide a detailed and segmented vision, facilitating the interpretation of the prevalence of IBS in different scenarios.

However, this review presents some limitations that should be considered when interpreting the results. First, the high heterogeneity (I^2^ > 90% in several analyses) indicates substantial differences in study methodologies (diagnostic criteria, type of sampling, sample size) and population characteristics (age, academic year, geographic region). Such diversity makes it difficult to estimate prevalence accurately and reduces investigation comparability. In addition, most of the included works employed cross-sectional designs, which prevents establishing causal relationships. Finally, studies based on non-probabilistic sampling could have affected the representativeness of the samples, limiting the generalization of the findings to the entire population of medical students.

Finally, another important limitation is that we did not extract or analyze data on IBS subtypes. This was not a pre-specified objective at the initiation of the review. Following peer review, we re-evaluated the included studies and found that fewer than five reported subtype distribution, with those that did often providing incomplete data or using inconsistent classification approaches. This paucity prevents meaningful meta-analysis of subtype-specific prevalence. The limited reporting of IBS subtypes in the primary literature is itself a notable finding, as subtypes have distinct pathophysiological mechanisms and treatment implications. The predominant subtype in medical students could provide insights into whether stress primarily affects gut motility in the context of constipation or diarrhea patterns, or whether dietary factors related to irregular academic schedules contribute differentially to subtype expression. Future primary studies should systematically report IBS subtype distribution according to Rome criteria, and future systematic reviews should include this as a key outcome to enable subtype-specific analyses.

## 5 Conclusions and recommendations

The findings of this SR and meta-analysis evidenced a significant prevalence of IBS in medical students, which varies according to diagnostic criteria (22% with Rome III vs. 17% with Rome IV), sampling methodology (higher in studies with probabilistic sampling), demographic characteristics (higher prevalence in women), and geographic region (ranging from 10% to 24% across WHO regions). However, high heterogeneity persisted across all subgroup analyses (I^2^ > 88% in most cases), indicating that variability reflects differences in local contextual factors–including population characteristics, cultural factors, and environmental contexts–rather than the broad categories examined. These results underscore the importance of IBS as a relevant health problem in the medical academic environment, with potential repercussions on educational performance and future clinical practice.

For future research, it is recommended to homogenize diagnostic criteria–employing Rome IV to a greater extent–and opt for probabilistic sampling methods that allow for more representative estimates. Likewise, it would be valuable to develop longitudinal studies to understand the progression of symptoms and evaluate the effectiveness of the proposed interventions, with special emphasis on managing academic stress and adopting healthy habits. Finally, implementing screening and comprehensive support programs within medical schools would contribute to early detection and care, minimizing the impact of IBS on the health and training of future doctors.

## Data Availability

The original contributions presented in this study are included in this article/Supplementary material, further inquiries can be directed to the corresponding author.
