# Supplementary material for: Prevalence of irritable bowel syndrome in medical students: a systematic review and meta-analysis
Source: Front Med (Lausanne). 2025 Dec 15;12:1714085. doi: 10.3389/fmed.2025.1714085 (PMC12745469; doi:10.3389/fmed.2025.1714085)
Supplement: Supplementary file 1 [file Table_1.DOCX]

Supplementary Material

**
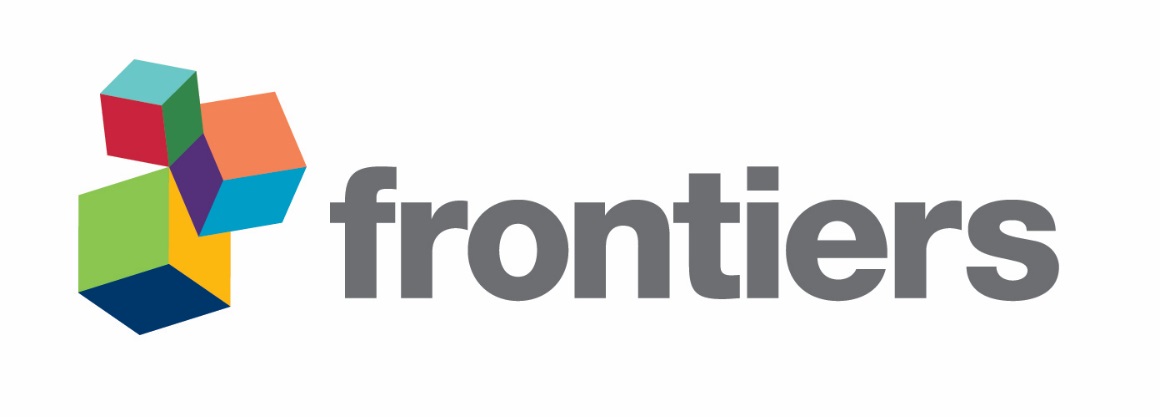
**

**Supplementary Material 1**. PRISMA 2020 Checklist

**Supplementary Material 2.** Search strategy

**Supplementary Material 3.** Main characteristics of the selected studies

**Supplementary Material 4.** Risk of Bias

**Supplementary Material 5.** Funnel plot of IBS prevalence according to Rome III (A) and Rome IV (B)

**Supplementary material 1**. PRISMA 2020 Checklist

| **Section and Topic** | **Item #** | **Checklist item** | **Location where item is reported** |
| --- | --- | --- | --- |
| **TITLE** | | |  |
| Title | 1 | Identify the report as a systematic review. | Page 1 |
| **ABSTRACT** | | |  |
| Abstract | 2 | See the PRISMA 2020 for Abstracts checklist. | Page 2 |
| **INTRODUCTION** | | |  |
| Rationale | 3 | Describe the rationale for the review in the context of existing knowledge. | Page 4 |
| Objectives | 4 | Provide an explicit statement of the objective(s) or question(s) the review addresses. | Page 4-5 |
| **METHODS** | | |  |
| Eligibility criteria | 5 | Specify the inclusion and exclusion criteria for the review and how studies were grouped for the syntheses. | Page 5 |
| Information sources | 6 | Specify all databases, registers, websites, organisations, reference lists and other sources searched or consulted to identify studies. Specify the date when each source was last searched or consulted. | Page 5 |
| Search strategy | 7 | Present the full search strategies for all databases, registers and websites, including any filters and limits used. | Page 6 |
| Selection process | 8 | Specify the methods used to decide whether a study met the inclusion criteria of the review, including how many reviewers screened each record and each report retrieved, whether they worked independently, and if applicable, details of automation tools used in the process. | Page 7 |
| Data collection process | 9 | Specify the methods used to collect data from reports, including how many reviewers collected data from each report, whether they worked independently, any processes for obtaining or confirming data from study investigators, and if applicable, details of automation tools used in the process. | Page 7 |
| Data items | 10a | List and define all outcomes for which data were sought. Specify whether all results that were compatible with each outcome domain in each study were sought (e.g. for all measures, time points, analyses), and if not, the methods used to decide which results to collect. | Page 8 |
|  | 10b | List and define all other variables for which data were sought (e.g. participant and intervention characteristics, funding sources). Describe any assumptions made about any missing or unclear information. | Page 8 |
| Study risk of bias assessment | 11 | Specify the methods used to assess risk of bias in the included studies, including details of the tool(s) used, how many reviewers assessed each study and whether they worked independently, and if applicable, details of automation tools used in the process. | Page 8 |
| Effect measures | 12 | Specify for each outcome the effect measure(s) (e.g. risk ratio, mean difference) used in the synthesis or presentation of results. | Page 8 |
| Synthesis methods | 13a | Describe the processes used to decide which studies were eligible for each synthesis (e.g. tabulating the study intervention characteristics and comparing against the planned groups for each synthesis (item #5)). | Page 8 |
|  | 13b | Describe any methods required to prepare the data for presentation or synthesis, such as handling of missing summary statistics, or data conversions. | Page 8 |
|  | 13c | Describe any methods used to tabulate or visually display results of individual studies and syntheses. | Page 8 |
|  | 13d | Describe any methods used to synthesize results and provide a rationale for the choice(s). If meta-analysis was performed, describe the model(s), method(s) to identify the presence and extent of statistical heterogeneity, and software package(s) used. | Page 8 |
|  | 13e | Describe any methods used to explore possible causes of heterogeneity among study results (e.g. subgroup analysis, meta-regression). | Page 8 |
|  | 13f | Describe any sensitivity analyses conducted to assess robustness of the synthesized results. | Page 8 |
| Reporting bias assessment | 14 | Describe any methods used to assess risk of bias due to missing results in a synthesis (arising from reporting biases). | ----- |
| Certainty assessment | 15 | Describe any methods used to assess certainty (or confidence) in the body of evidence for an outcome. | ----- |
| **RESULTS** | | |  |
| Study selection | 16a | Describe the results of the search and selection process, from the number of records identified in the search to the number of studies included in the review, ideally using a flow diagram. | Page 8 |
|  | 16b | Cite studies that might appear to meet the inclusion criteria, but which were excluded, and explain why they were excluded. | Page 8 |
| Study characteristics | 17 | Cite each included study and present its characteristics. | Page 8 |
| Risk of bias in studies | 18 | Present assessments of risk of bias for each included study. | Page 8-9 |
| Results of individual studies | 19 | For all outcomes, present, for each study: (a) summary statistics for each group (where appropriate) and (b) an effect estimate and its precision (e.g. confidence/credible interval), ideally using structured tables or plots. | Page 8-9 |
| Results of syntheses | 20a | For each synthesis, briefly summarise the characteristics and risk of bias among contributing studies. | Page 8-9 |
|  | 20b | Present results of all statistical syntheses conducted. If meta-analysis was done, present for each the summary estimate and its precision (e.g. confidence/credible interval) and measures of statistical heterogeneity. If comparing groups, describe the direction of the effect. | Page 8-9 |
|  | 20c | Present results of all investigations of possible causes of heterogeneity among study results. | Page 8-9 |
|  | 20d | Present results of all sensitivity analyses conducted to assess the robustness of the synthesized results. | Page 8-9 |
| Reporting biases | 21 | Present assessments of risk of bias due to missing results (arising from reporting biases) for each synthesis assessed. | Page 8-9 |
| Certainty of evidence | 22 | Present assessments of certainty (or confidence) in the body of evidence for each outcome assessed. | Page 9 |
| **DISCUSSION** | | |  |
| Discussion | 23a | Provide a general interpretation of the results in the context of other evidence. | Page 10 |
|  | 23b | Discuss any limitations of the evidence included in the review. | Page 11-12 |
|  | 23c | Discuss any limitations of the review processes used. | Page 11-12 |
|  | 23d | Discuss implications of the results for practice, policy, and future research. | Page 11-12 |
| **OTHER INFORMATION** | | |  |
| Registration and protocol | 24a | Provide registration information for the review, including register name and registration number, or state that the review was not registered. | Page 6 |
|  | 24b | Indicate where the review protocol can be accessed, or state that a protocol was not prepared. | Page 6 |
|  | 24c | Describe and explain any amendments to information provided at registration or in the protocol. | Page 6 |
| Support | 25 | Describe sources of financial or non-financial support for the review, and the role of the funders or sponsors in the review. | Page 1 |
| Competing interests | 26 | Declare any competing interests of review authors. | Page 6 |
| Availability of data, code and other materials | 27 | Report which of the following are publicly available and where they can be found: template data collection forms; data extracted from included studies; data used for all analyses; analytic code; any other materials used in the review. | Page 6 |

From: Page MJ, McKenzie JE, Bossuyt PM, Boutron I, Hoffmann TC, Mulrow CD, et al. The PRISMA 2020 statement: an updated guideline for reporting systematic reviews. BMJ 2021;372:n71. doi: 10.1136/bmj.n71. This work is licensed under CC BY 4.0. To view a copy of this license, visit <https://creativecommons.org/licenses/by/4.0/>

**Supplementary material 2**. Search strategy

| Search strategy in PUBMED | |
| --- | --- |
| #1 | "Irritable Bowel Syndrome"[Mesh] OR "irritable bowel syndrome"[tiab] OR IBS[tiab] OR "síndrome de intestino irritable"[tiab] OR "sindrome de intestino irritable"[tiab] OR "colon irritable"[tiab] OR "colón irritable"[tiab] OR "spastic colon"[tiab] OR "mucous colitis"[tiab] OR "colitis mucosa"[tiab] |
| #2 | "Students, Medical"[Mesh] OR "medical student"[tiab] OR "medical students"[tiab] OR "estudiante de medicina"[tiab] OR "estudiantes de medicina"[tiab] |
| #3 | #1 AND #2 AND #3 |
| Search strategy in SCOPUS | |
| #1 | TITLE-ABS-KEY( "Irritable Bowel Syndrome" OR "irritable bowel syndrome" OR IBS OR "síndrome de intestino irritable" OR "sindrome de intestino irritable" OR "colon irritable" OR "colón irritable" OR "spastic colon" OR "mucous colitis" OR "colitis mucosa" ) |
| #2 | TITLE-ABS-KEY( "Students, Medical" OR "medical student" OR "medical students" OR "estudiante de medicina" OR "estudiantes de medicina" ) |
| #3 | #1 AND #2 AND #3 |
| Search strategy in Web of Science | |
| #1 | TS=("Irritable Bowel Syndrome" OR "irritable bowel syndrome" OR IBS OR "síndrome de intestino irritable" OR "sindrome de intestino irritable" OR "colon irritable" OR "colón irritable" OR "spastic colon" OR "mucous colitis" OR "colitis mucosa") |
| #2 | TS=("Students, Medical" OR "medical student" OR "medical students" OR "estudiante de medicina" OR "estudiantes de medicina") |
| #4 | #1 AND #2 |
| Search strategy in EMBASE | |
| #1 | ('irritable colon'/exp OR 'colon spasm' OR 'colon, irritable' OR 'colonic diseases, functional' OR 'colonospasm' OR 'functional colonic diseases' OR 'irritable bowel syndrome' OR 'irritable colon' OR 'irritable colon syndrome' OR 'mucomembraneous colitis' OR 'mucomembranous colitis' OR 'mucous colitis' OR 'spastic colitis' OR 'spastic colon' OR 'unstable colon') |
| #2 | ('medical student'/exp OR 'med school student' OR 'med student' OR 'medical school student' OR 'medical student' OR 'medical students' OR 'student, medical' OR 'students, medical') |
| #4 | #1 AND #2 |

**Supplementary Material 3**. Main characteristics of the selected studies

| **First author** | **Year** | **Country** | **Study type** | **Sampling** | **Selection criteria** | **Sample** | **Sex (% women)** | **Age (average or median)** |
| --- | --- | --- | --- | --- | --- | --- | --- | --- |
| Jung | 2011 | Korea | Cross-sectional | Non-probabilistic | Medical students in Busan, Korea. | 319 | 80 (25.07%) | 22.3 |
| Wells | 2012 | Canada | Cross-sectional | Non-probabilistic | Preclinical and clinical medical students in Ontario. Excluded: pre-existing gastrointestinal disorders. | 228 | 104 (47.7%) | 25 |
| Naeem | 2012 | Pakistan | Cross-sectional | Non-probabilistic | Medical students from all years of three faculties >18 years with informed consent. | 360 | 297 | __ |
| Basandra | 2014 | India | Cross-sectional | Non-probabilistic | Medical students from VI semester. Excluded: people with intellectual disabilities. | 200 | 110 (55%) | 20.43 |
| Liu | 2014 | China | Cross-sectional | Probabilistic | Medical students from 1st to 7th year. Excluded: organic gastrointestinal disorders, family history of cancer, alarm signs or previous gastrointestinal surgery. | 767 | 571 (74.4%) | 23.26 |
| Vargas-Matos | 2015 | Peru | Cross-sectional | Non-probabilistic | Medical students. Excluded: conditions that could confuse diagnosis of IBS and dyspepsia. | 380 | 222 (58.58%) | 19 |
| Perveen | 2016 | Bangladesh | Cross-sectional | Non-probabilistic | Students from 4 medical schools with informed consent. | 293 | 116 (39.59%) | 21.09 |
| Wang | 2016 | China | Cross-sectional | Non-probabilistic | Undergraduate university students of medicine and other sciences. | 1874 | 1342 (71.61%) | __ |
| Alaqeel | 2017 | Saudi Arabia | Cross-sectional | Non-probabilistic | Medical students from 1st to 5th year (2015-2016). | 270 | 88 | __ |
| Pozos-Rodillo | 2018 | Mexico | Cross-sectional | Probabilistic | Medical university students in Guadalajara, Mexico. | 561 | 293 (52%) | 20.97 |
| Sehonou | 2018 | Benin | Cross-sectional | Non-probabilistic | Medical students from 2nd to 6th year. Excluded: organic gastrointestinal or gynecological pathologies and first-year students. | 315 | 133 (42.2%) | 21.9 |
| Elhosseiny | 2019 | Egypt | Cross-sectional | Probabilistic | Medical students from 1st to 6th year (18-25 years, 2017-2018). Excluded: organic gastrointestinal disorders or alarm symptoms. | 382 | 132 (34.55%) | 20.69 |
| Vasquez-Rios | 2019 | Peru | Cross-sectional | Non-probabilistic | Full-time students ≥18 years from 4th to 7th year of medicine. Excluded: known gastrointestinal disorders and pregnant women. | 346 | 162 (46.82%) | __ |
| Eltayeb | 2020 | Sudan | Cross-sectional | Probabilistic | Medical student from Al-Neelain University. | 121 | 54 (44.62%) | 22 |
| Seger | 2020 | Malaysia | Cross-sectional | Non-probabilistic | Medical students from 6th to 9th semester at a private university in Malaysia (Aug-Dec 2018). Excluded: previous diagnosis of IBS. | 190 | 107 (56.3%) | 23.18 |
| Al-Mutori | 2020 | Oman | Cross-sectional | Non-probabilistic | Medical students from the National University of Science and Technology in Oman (Jun-Jul 2017). | 464 | 390 (84.05%) | __ |
| Tauseef | 2021 | Pakistan | Cross-sectional | Non-probabilistic | Medical students from 1st to 5th year of Allama Iqbal Medical College (2020-2021). Excluded: students with gastrointestinal alarm symptoms. | 322 | 213 (66.14%) | ___ |
| Anthea | 2021 | Malta | Cross-sectional | Non-probabilistic | Medical students from the University of Malta. | 135 | __ | __ |
| Ramírez-Amill | 2021 | Puerto Rico | Cross-sectional | Non-probabilistic | Medical students from 1st to 4th year from the University of Puerto Rico. | 314 | 151 (48.1%) | 24.8 |
| Alreshidi | 2022 | Saudi Arabia | Cross-sectional | Non-probabilistic | Medical students from the University of Ha'il (Nov 2021-Feb 2022). | 308 | 134 (43.5%) | __ |
| Maghoudi | 2022 | Iran | Cross-sectional | Probabilistic | Medical students from Isfahan Medical Sciences (Jan 2018-Mar 2019). Excluded: students with gastrointestinal alarm signs, university dropouts, or family history of gastric cancer. | 100 | 68 (68%) | 23.2 |
| Alfaqih | 2022 | Saudi Arabia | Cross-sectional | Non-probabilistic | Medical students from 2nd to 6th year of AlQufudah faculty (Jan-Mar 2022). Excluded: incomplete responses. | 270 | 141 (52.2%) | ___ |
| Mirghani | 2022 | Saudi Arabia | Cross-sectional | Non-probabilistic | Medical students from 2nd to 6th year and interns from the University of Tabuk. Excluded: first-year students. | 321 | 195 (60.7%) | __ |
| Fadl | 2022 | Saudi Arabia | Cross-sectional | Non-probabilistic | Medical students from Saudi universities. Excluded: postgraduate students, non-medical or from other universities. | 300 | 191 (63.7%) | __ |
| Al-Zahrani | 2022 | Saudi Arabia | Cross-sectional | Non-probabilistic | Medical students from 2nd to 6th year from Umm Al-Qura University. | 303 | 123 (40.6%) | __ |
| Gallas | 2022 | Tunisia | Cross-sectional | Non-probabilistic | Students from 1st and 2nd year of Medicine (Feb-Mar 2015). Excluded: ulcerative disease, IBD or abdominal surgery (except appendectomy). | 343 | 235 (68.5%) | 20.3 |
| Jadallah | 2022 | Jordan | Cross-sectional | Non-probabilistic | Medical students from 1st to 6th year (Jan-Apr 2020). Excluded: significant comorbidities, IBD, celiac disease, lactose intolerance or ulcerative disease. | 1094 | 594 (54.3%) | __ |
| Jia | 2022 | China | Cross-sectional | Probabilistic | Students from all years of medicine with consent. Excluded: other gastrointestinal conditions, neoplasms, extraintestinal disease, alarm signs, substance abuse, dysmenorrhea. | 2739 | 1021 | __ |
| Farah | 2022 | Jordan | Cross-sectional | Non-probabilistic | Medical students from the University of Jordan. Excluded: history of IBD, celiac disease, diabetes, undiagnosed allergy or duodenal ulcer. | 558 | 227 (40.7%) | __ |
| Javed | 2022 | Pakistan | Cross-sectional | Non-probabilistic | Medical students >18 years from all academic years. Excluded: non-medical students and diagnosed cases of gastrointestinal disease. | 305 | 120 (39.30%) | 21.43 |
| Cadavid-López | 2023 | Colombia | Cross-sectional | Non-probabilistic | Medical students from San Martin University Foundation (2016). Excluded: previous diagnosis of organic gastrointestinal disorders, current treatment for gastrointestinal symptoms, alarm signs. | 173 | 119 (68.79%) | 22 |
| Tran | 2023 | Malaysia | Cross-sectional | Non-probabilistic | Medical students >18 years who completed questionnaire. Excluded: missing data or without urine sample. | 400 | 152 (38%) | 19 |
| Mujamammi | 2023 | Saudi Arabia | Cross-sectional | Probabilistic | Medical students from King Saud University (Nov-Dec 2020). Excluded: without consent, diabetes, celiac disease, IBD, gastrointestinal cancer or current infection. | 426 | 155 (36.38%) | 21.21 |
| Valladares-Garrido | 2024 | Peru | Cross-sectional | Non-probabilistic | Medical students from 1st to 7th year from San Martín de Porres University (2021-II). Excluded: without consent, previous IBS diagnosis, gastrointestinal pathologies or recent surgery. | 137 | 0% | 22 |
| Liptak | 2024 | Slovakia | Cross-sectional | Non-probabilistic | Medical students >18 years from 3 universities. Excluded: gastrointestinal alarm signs, specific digestive disorders or family history of IBD/cancer. | 1061 | 757 (71.3%) | 22.6 years |
| Das | 2024 | Bangladesh | Cross-sectional | Non-probabilistic | Medical students residing in Bangladesh. | 402 | 229 (56.97%) | ___ |
| Park | 2024 | Korea | Cross-sectional | Non-probabilistic | Korean adults from medical school. Excluded: participants with organic gastrointestinal diseases. | 338 | 163 (48.2%) | 21.9 |
| Alshehri | 2024 | Saudi Arabia | Cross-sectional | Probabilistic | Medical students from 2nd to 6th year. | 363 | 171 (47.1%) | 22.1 |
| Mahyoub | 2024 | Yemen | Cross-sectional | Non-probabilistic | Medical students from 1st to 6th year from Ibb University with consent. Excluded: abdominal pain related to menstrual cycle, organic gastrointestinal disorders, alarm signs. | 351 | 139 (39.60%) | 22.53 |
| Medina-Pérez | 2024 | Peru | Cross-sectional | Non-probabilistic | Adult medical students (5th-7th year) without previous gastrointestinal diagnosis. Excluded: risk factors such as cancer history, bleeding or alarm signs. | 131 | 64 (48.85%) | 24.04 |
| Frere | 2024 | Egypt | Cross-sectional | Probabilistic | Students from 1st to 5th year of Medicine. Excluded: organic gastrointestinal disorders, previous surgery, chronic diseases or psychiatric disorders. | 221 | 127 (57.5%) | 20.2 |
| Quiroga-Castañeda | 2024 | Peru | Cross-sectional | Probabilistic | Medical students from 1st to 7th year (2021-2022). Excluded: refusal to participate, previous IBS diagnosis, recent gastrointestinal pathologies or surgeries. | 409 | 272 (66.5%) | 21 |
| Fraij | 2024 | United Arab Emirates | Cross-sectional | Probabilistic | Undergraduate students of medicine and other health faculties. Excluded: pre-existing gastrointestinal disorders or alarm signs. | 224 | __ | 20.1 |

**Supplemental Material 5.** Risk of Bias

| **Study, year** | **1** | **2** | **3** | **4** | **5** | **6** | **7** | **8** | **9** | **Total** | **Risk of Bias** |
| --- | --- | --- | --- | --- | --- | --- | --- | --- | --- | --- | --- |
| Jung 2011 | Y | N | Y | Y | Y | Y | Y | Y | N | 7 | Low |
| Wells 2012 | Y | N | Y | Y | Y | Y | Y | Y | N | 7 | Low |
| Naeem 2012 | Y | N | Y | Y | Y | Y | Y | Y | N | 7 | Low |
| Basandra 2014 | Y | N | Y | Y | Y | Y | Y | Y | N | 7 | Low |
| Liu 2014 | Y | Y | Y | Y | Y | Y | Y | Y | N | 8 | Low |
| Vargas-Matos 2015 | Y | N | Y | Y | Y | Y | Y | Y | N | 7 | Low |
| Perveen 2016 | Y | N | Y | Y | Y | Y | Y | Y | N | 7 | Low |
| Wang 2016 | Y | N | Y | Y | Y | Y | Y | Y | N | 7 | Low |
| Alaqeel 2017 | Y | N | Y | Y | Y | Y | Y | Y | N | 7 | Low |
| Pozos-Rodillo 2018 | Y | Y | Y | Y | Y | Y | Y | Y | N | 8 | Low |
| Sehonou 2018 | Y | N | Y | Y | Y | Y | Y | Y | N | 7 | Low |
| Elhosseiny 2019 | Y | Y | Y | Y | Y | Y | Y | Y | N | 8 | Low |
| Vasquez-Rios 2019 | Y | N | Y | Y | Y | Y | Y | Y | N | 7 | Low |
| Eltayeb 2020 | Y | Y | Y | Y | Y | Y | Y | Y | N | 8 | Low |
| Seger 2020 | Y | N | Y | Y | Y | Y | Y | Y | N | 7 | Low |
| Al-Mutori 2020 | Y | N | Y | Y | Y | Y | Y | Y | N | 7 | Low |
| Tauseef 2021 | Y | N | Y | Y | Y | Y | Y | Y | N | 7 | Low |
| Anthea 2021 | Y | N | Y | Y | Y | Y | Y | Y | N | 7 | Low |
| Ramirez-Amill 2021 | Y | N | Y | Y | Y | Y | Y | Y | N | 7 | Low |
| Alreshidi 2022 | Y | N | Y | Y | Y | Y | Y | Y | N | 7 | Low |
| Maghoudi 2022 | Y | Y | Y | Y | Y | Y | Y | Y | N | 8 | Low |
| Alfaqih 2022 | Y | N | Y | Y | Y | Y | Y | Y | N | 7 | Low |
| Mirghani 2022 | Y | N | Y | Y | Y | Y | Y | Y | N | 7 | Low |
| Fadl 2022 | Y | N | Y | Y | Y | Y | Y | Y | N | 7 | Low |
| Al-Zahrani 2022 | Y | N | Y | Y | Y | Y | Y | Y | N | 7 | Low |
| Gallas 2022 | Y | N | Y | Y | Y | Y | Y | Y | N | 7 | Low |
| Jadallah 2022 | Y | N | Y | Y | Y | Y | Y | Y | N | 7 | Low |
| Jia 2022 | Y | Y | Y | Y | Y | Y | Y | Y | N | 8 | Low |
| Farah 2022 | Y | N | Y | Y | Y | Y | Y | Y | N | 7 | Low |
| Javed 2022 | Y | N | Y | Y | Y | Y | Y | Y | N | 7 | Low |
| Cadavid-Lopez 2023 | Y | N | Y | Y | Y | Y | Y | Y | N | 7 | Low |
| Tran 2023 | Y | N | Y | Y | Y | Y | Y | Y | N | 7 | Low |
| Mujamammi 2023 | Y | Y | Y | Y | Y | Y | Y | Y | N | 8 | Low |
| Valladares-Garrido 2024 | Y | N | Y | Y | Y | Y | Y | Y | N | 7 | Low |
| Liptak 2024 | Y | N | Y | Y | Y | Y | Y | Y | N | 7 | Low |
| Das 2024 | Y | N | Y | Y | Y | Y | Y | Y | N | 7 | Low |
| Park 2024 | Y | N | Y | Y | Y | Y | Y | Y | N | 7 | Low |
| Alshehri 2024 | Y | Y | Y | Y | Y | Y | Y | Y | N | 8 | Low |
| Mahyoub 2024 | Y | N | Y | Y | Y | Y | Y | Y | N | 7 | Low |
| Medina-Pérez 2024 | Y | N | Y | Y | Y | Y | Y | Y | N | 7 | Low |
| Frere 2024 | Y | Y | Y | Y | Y | Y | Y | Y | N | 8 | Low |
| Quiroga-Castañeda 2024 | Y | Y | Y | Y | Y | Y | Y | Y | N | 8 | Low |
| Fraij 2024 | Y | Y | Y | Y | Y | Y | Y | Y | N | 8 | Low |

1. Was the sample frame appropriate to address the target population? 2. Were study participants recruited in an appropriate way? 3. Was the sample size adequate? 4. Were the study subjects and setting described in detail? 5. Was data analysis conducted with sufficient coverage of the identified sample? 6. Were valid methods used for the identification of the condition? 7. Was the condition measured in a standard, reliable way for all participants? 8. Was there appropriate statistical analysis? 9. Was the response rate adequate, and if not, was the low response rate managed appropriately. Y = Yes, N = No, U = Unclear


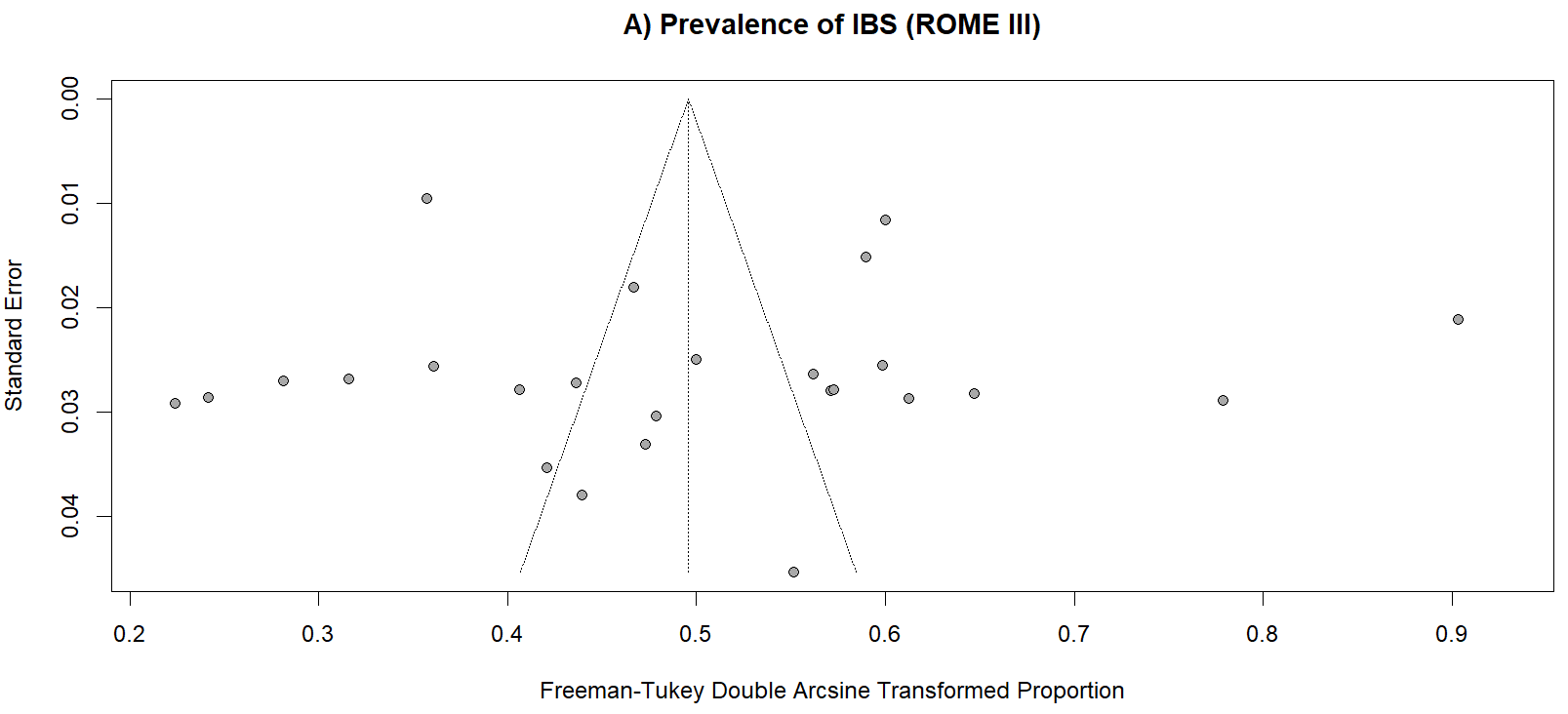

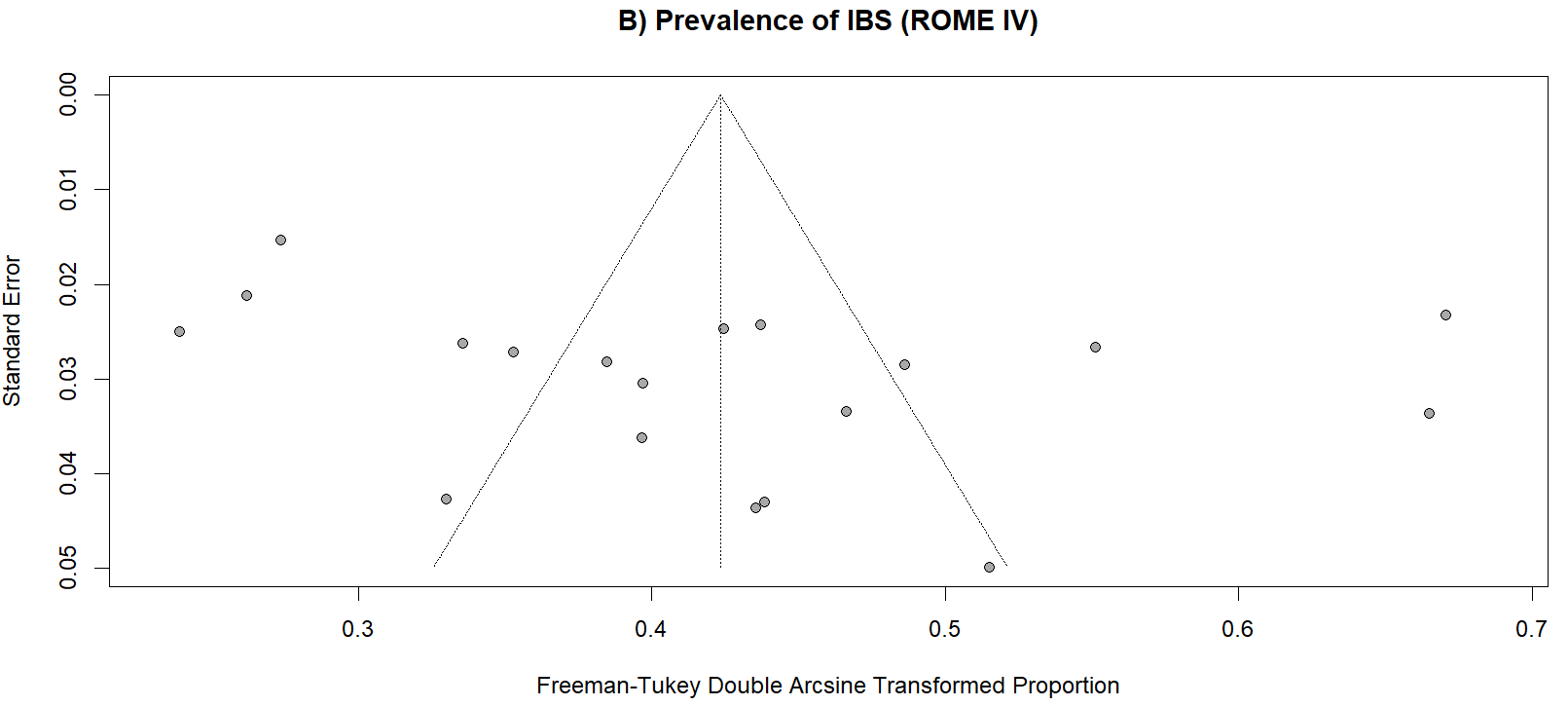


**Supplementary material 5**. Funnel plot of IBS prevalence according to Rome III (A) and Rome IV (B)
